# Molecular analyses of ADAMTS-1, -4, -5, and IL-17 a cytokine relationship in patients with ulcerative colitis

**DOI:** 10.1186/s12876-023-02985-z

**Published:** 2023-10-05

**Authors:** Tahir Buran, Muhammet Burak Batır, Fethi Sırrı Çam, Elmas Kasap, Fatih Çöllü, Hamide Betül Gerik Çelebi, Mustafa Şahin

**Affiliations:** 1 Department of Gastroenterology, Faculty of Medicine, Manisa Celal Bayar University, Manisa, Turkey; 2 Department of Biology, Faculty of Arts and Sciences, Manisa Celal Bayar University, Manisa, Turkey; 3 Department of Medical Genetics, Faculty of Medicine, Manisa Celal Bayar University, Manisa, Turkey; 4 Department of Medical Genetics, Atatürk City Hospital, Balıkesir, Turkey; 5 Department of Internal Medicine, Faculty of Medicine, Manisa Celal Bayar University, Manisa, Turkey

**Keywords:** Ulcerative colitis, IL-17A, ADAMTS, Inflammation

## Abstract

**Background:**

Ulcerative colitis (UC) is a chronic inflammatory bowel disease that develops due to the impaired immune response in genetically susceptible individuals, and its etiopathogenesis is not fully elucidated. IL-17 A is a cytokine that is produced by a type of immune cell called Th17 cells and is involved in the immune response and inflammation. On the other hand, ADAMTS-1, -4, and − 5 are enzymes that are involved in the breakdown of extracellular matrix proteins, including proteoglycans, which are important components of the intestinal wall. This study aimed to evaluate the relationship between interleukin 17 (IL-17 A) cytokine, which plays a role in the pathogenesis of ulcerative colitis, and the inflammation-controlled a disintegrin and metalloproteinase with thrombospondin motifs (ADAMTS)-1, -4, and − 5 protein members.

**Methods:**

Bowel tissue samples and blood serum from 51 patients with UC and 51 healthy controls were included in this study. mRNA expression levels of the ADAMTS-1, -4, -5, and IL-17 A were analyzed by RT-qPCR, and immunohistochemical analyses were performed to evaluate ADAMTS-1, -4, -5, and IL-17 A proteins in tissue samples. In addition, ELISA analysis determined serum levels of the ADAMTS-1, -4, -5, and IL-17 A.

**Results:**

RT-qPCR results reveal that the expression of ADAMTS-1, -4, -5, and IL-17 A genes in the UC tissue samples were significantly high according to the control tissue samples. Also, ADAMTS-1, -4, -5, and IL-17 A proteins revealed enhanced expression pattern UC groups according to the control. Also, ADAMTS-1, -4, -5, and IL-17 A protein showed cytoplasmic localization patterns in both control and UC groups. The serum levels of ADAMTS-1,-5, and IL-17 A were significantly higher in UC samples than in the control group.

**Conclusions:**

We observed a positive correlation between the ADAMTS-1, -5 and IL17A cytokine expression in UC samples. These results provide a new understanding of controlling crucial ADAMTS family protein members by IL-17 A cytokines with UC.

## Introduction

Ulcerative colitis (UC) appears on the background of abnormal mucosal immune response and progresses with relapses in the bowel [1]. UC is limited to the colon, starting from the rectum and spreading toward the proximal colon, and is characterized by ulcerations in the mucosa and submucosa [2, 3]. Even though UC can arise at any age, it is the most common in the group the age of 15–30 years, followed by the group of the age of 50–70 years [2]. Although the etiopathogenesis of UC has not been fully elucidated yet, the combination of environmental, genetic, and immune regulatory factors has been hypothesized [4, 5]. Risk factors such as infectious agents, drugs, diet, and stress constitute an important predisposition to UC. The leading current hypothesis in the aetiology of inflammatory bowel disease (IBD) highlights genetic predisposition leading to the dysregulated gastrointestinal and immune systems [6].

The immune system mechanisms could vary in UC. Therefore, the cellular immune response may play a role in the pathogenesis of IBD. T lymphocytes provide cellular immunity; these are functionally classified as CD4 + T helper cells, CD8 (cytotoxic), and regulatory T (Treg) cells. CD + T helper cells are functionally categorized into three subgroups Th1, Th2, and Th17 [7]. While, Th1 cells secrete interferon-gamma (IFN gamma), tumour necrosis factor-alpha, interleukin-2, and interleukin-12, the Th2 cells primarily regulate B cell differentiation by secreting IL-4, IL-5, and IL-13. On the other hand, Th17 cells play a critical role in regulating inflammatory processes and may have a role in autoimmunity. Also, Th17 cells predominantly secrete interleukin (IL)-17, IL-6, and GCSF [8].

IL-17 A is a cytokine produced by a type of immune cell called Th17 cells, which are involved in the immune response to pathogens and play a role in autoimmune diseases like UC [9–11]. In addition, IL-17 A triggers the production of other cytokines and chemokines, promoting inflammation and recruiting immune cells to sites of infection or inflammation. Research revealed that individuals with UC have elevated levels of IL-17 A in their inflamed tissues [12–14].

Thrombospondin motifs (ADAMTS) family are associated with A disintegrin and metalloproteinase, which are involved in the construction of the extracellular matrix and the pathogenesis of many chronic inflammations such as atherosclerosis and osteoarthritis [15]. These enzymes released in the extracellular matrix also have roles in many physiological events, such as the production and destruction of the matrix, the rearrangement of tissues, coagulation, and fibrosis. Also, recent researches have suggested that there may be a relationship between IL-17 A and ADAMTS. A study revealed that the IL-17 A synergistically induced the expressions of ADAMTS-1, -4, and − 5 enzymes [9]. Also, another study found that treating rats with an inhibitor of IL-17 A signaling reduced the expression of ADAMTS-5 in their inflamed joint tissue [16].

Unfortunately, the action of IL-17 A cytokines on the expression of the ADAMTS family in UC pathogenesis is poorly understood. For this reason, the objective of this study was to determine the action of IL-17 A on the expression of ADAMTS-1, -4 and − 5 in UC samples. The data obtained from this study will make novel contributions to the literature on the pathogenesis of UC related to IL-17 A and ADAMTS.

## Materials and methods

A total of 1312 patients who underwent colonoscopy between April 2016 and May 2020 in the Gastroenterology Clinic of Celal Bayar University Medical Faculty Hospital, 51 patients over 18 years old diagnosed histopathologically with UC, were included. As control cases, 51 healthy people found to be normal after colon scanning were included. The exclusion criteria were concomitant colon stenosis, colon tumours, patients who underwent rectosigmoidescopy without a complete colonoscopy, those with colon pathology other than ulcerative colitis (diverticulitis, ischemic colitis, radiation colitis, Crohn’s disease), and those with concomitant chronic diseases (such as rheumatoid arthritis, autoimmune diseases). The study was approved by the Clinical Research Ethics Committee of Manisa Celal Bayar University (Manisa, Turkiye), and written informed consent was obtained from all subjects.

### Total RNA extraction and real-time quantitative PCR (RT-qPCR)

Total RNA from colon samples was extracted using TRIzol® Reagent with the PureLink® RNA Mini Kit (Thermo Fisher Scientific, 12183555). cDNA was synthesized using the extracted RNA samples according to the High-Capacity cDNA Reverse Transcription Kit (AppliedBiosystems, 4368814) procedure in a thermal cycler (SensoQuest, Gottingen, Germany). On the other hand, RT-PCR from the cDNA was performed using primers of ADAMTS-1, ADAMTS-4, ADAMTS-5, and IL-17A and Fast Evagreen qPCR Master Mix (Biotium, 31003). The master mixes for each primer were separately prepared primer for each target gene, and qPCR was conducted in the Rotor-Gene Q (Qiagen, Hilden, Germany) to evaluate expression levels of ADAMTS-1, ADAMTS-4, ADAMTS-5, and IL-17A genes. Hypoxanthine phosphoribosyltransferase (HPRT1) was used as a housekeeping gene for normalizing the expression changes. The related forward and reverse primers were ADAMTS1F 5’ CAAGCCTCAGAATCCCATA 3’, ADAMTS1R 5’ TCCTCCCCAAATGTAAACT 3’, ADAMTS4F 5’ CCTCTCCCATGACGATTC 3’, ADAMTS4R 5’ TTGAAGTCCTGGAGCTGGT 3’, ADAMTS5F 5’ CCTCTCCCATGACGATTC 3’, ADAMTS5R 5’ CTGTGATGGTGGCTGAAG 3’, IL17F 5’ ACAATCCCACGAAAT 3’, IL17R 5’ AAGGTGAGGTGGATC 3’, HPRTF 5’ CGTCTTGCTCGAGAT 3’, HPRTR 5’ TTCAGTGCTTTGATGTA 3’. The cycling condition of the RT-qPCR was started with an initial activation/denaturation step at 94 °C (15 min), followed by 35 cycles of denaturation at 95 °C for 20 s, and a combined annealing/extension step at 59 °C for 50 s. The 2^−ΔΔCT^ procedure was used to evaluate the relative changes in gene expression [17].

### Enzyme-linked Immunosorbent Assay (ELISA)

Peripheral venous blood specimens (2 ccs) of the analysis were put into serum separator tubes containing a gel matrix that serves as a barrier between the blood cells and the serum to receive serum for ELISA. The tube was centrifuged at 2000 rpm for 15 min. The supernatant was stored at − 80 °C. Human ELISA Kit (MyBioSource, Inc., CA, USA) was used for quantitative measure of ADAMTS-1, ADAMTS-4, ADAMTS-5, and IL-17 A serum levels.

### Immunohistochemistry (IHC)

The colon specimens were fixed in a 10% phosphate-buffered formalin solution for 30 h. Following the dehydration procedure, the colon samples were embedded in paraffin, and the prepared paraffin blocks were dissected at a 5 mm thickness. After being deparaffinized and rehydration in graded ethanol series, sections were rinsed. Next, rinsed sections were treated with the 4% H2O2 for 10 min to bleach the endogenous peroxidases and were then rinsed twice in phosphate-buffered saline (PBS) for 5 min. The sections were then set in 10X citrate buffer for epitope retrieval in a water bath at 96 °C for 30 min, followed by rinsing thrice in PBS for 5 min. Subsequently, prepared sections were treated with blocking solution (EMD Millipore IHC Select Blocking Reagent, 20,773) for 30 min and blocked sections were then separately incubated overnight with ADAMTS-1 antibody (1:100 PBS diluted; Santa Cruz Biotechnology-47,726), ADAMTS-4 antibody (Novus 416,610), ADAMTS-5 antibody (Novus 362,810), and IL-17 A antibody (Novus 41,809) monoclonal antibodies at + 4 °C. After incubation, PBS-wash was given three times, and the sections were then incubated with the 1:200 PBS diluted biotinylated chicken secondary anti-mouse antibody (Abcam, ab6813) for 30 min. After the incubation step, sections were rinsed two times for 10 min with PBS and stained with 3.3-diaminobenzidine tetrahydrochloride (DAB Staining Kit, Vectorlab, SK-4100) counterstained with hematoxylin. Eventually, the sections were dehydrated and immersed in xylene, and coverslips were placed on them. All images were evaluated by light microscopy using a 20X objective (Olympus BX43). Positive staining was scored using the Image-Pro Plus v6.0 software to calculate the percentage (%) of the optical density (OD) value of DAB-stained tissue surface sites [18].

### Statistical analysis

Data are given as a mean ± standard deviation (SD). The SPSS (statistical package software, Windows 23.0) was used to analyze the significance of differences observed between the groups. RT-PCR, ELISA, and IHC values were found to be abnormally distributed. Hence, the data obtained from RT-PCR, ELISA, and IHC analysis were tested by performing Independent Samples t Test. *p* < 0.05 was considered as statistically significant [19].

## Results

The age of male patients included in this study was 31years (60.79%), that of female patients was 20 years (39.21%), and the average age of the patients was 49.04 years. Besides, the age of healthy male controls was 28 years (54.90%), that of healthy female controls was 23 (45.10%), and the average age of control participants was 57.22 years (Table 1). Also, there were no individuals with cardiovascular disease, diabetes mellitus and other chronic diseases in either group.

**Table 1:** Demographics of patients with ulcerative colitis and the healthy control group

	Ulcerative colitis patients (n = 51)	%	Control group (n = 51)	%	
Gender					
Male Woman	31 20	60.79 39.21	28 23	54.90 45.10	
Age(years)(average) Male Woman	49.04 54.57 (34–77) 39.94 (21–69)		57.22 58.15 (38–81) 55.94 (34–75)		
Cardiovascular disease (n)	0		0		
Hypertension (n)	0		0		
Diabetes mellitus (n)	0		0		
Other chronic diseases (n)	0		0		
Smoking	4		7		
	**Disease extension in ulcerative colitis patients**	%	**Disease extension in control group**		%
Ulcerative proctitis (n)	21	41.2	N/A		-
Left-sided colitis (n)	12	23.5	N/A		-
Extensive colitis (n)	18	35.3	N/A		-
	**Disease Severity in ulcerative colitis patients**	%	**Disease Severity in control group**		%
Mild (n)	25	49.0	N/A		-
Moderate (n)	14	27.5	N/A		-
Severe (n)	12	23.5	N/A		-

### Gene expression level of the ADAMTS-1, ADAMTS-4, ADAMTS-5 and IL-17 A

**Fig. 1:**
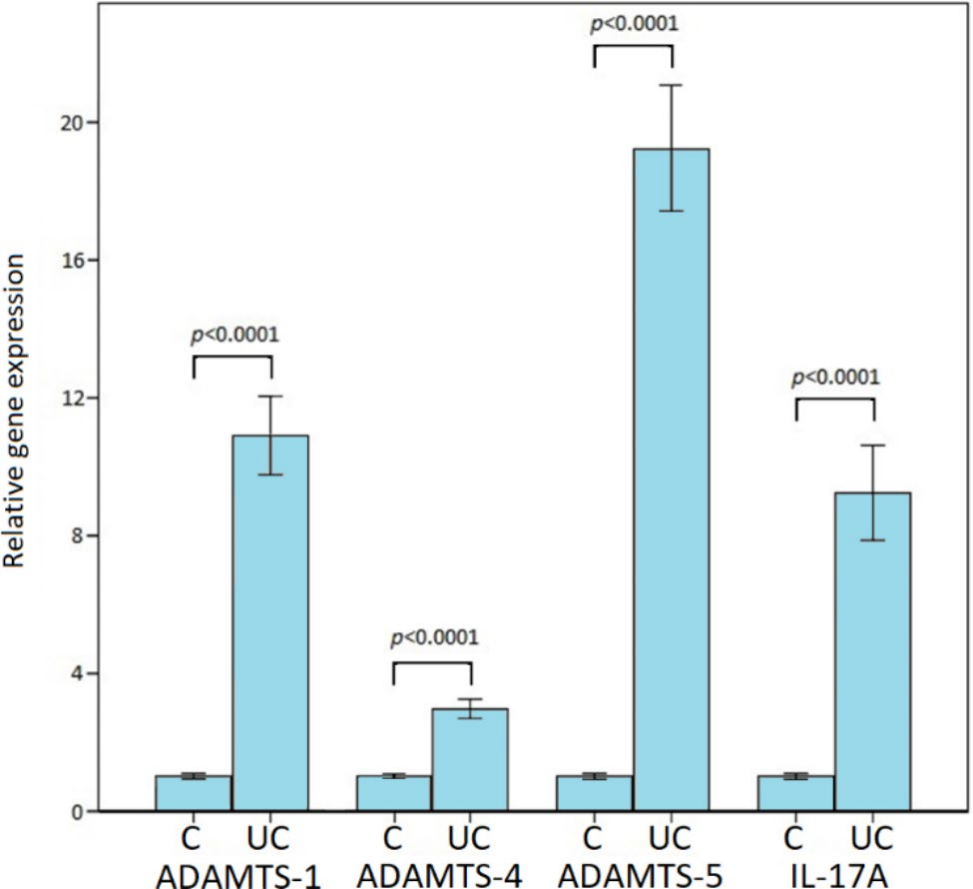
RT-PCR analysis of the ADAMTS-1, -4, -5, and IL-17 A in the healthy human control (C) and colon tissues with ulcerative colitis (UC). Values (fold change) are represented as mean ± standard deviation

RT-qPCR was used to detect ADAMTS-1, -4, -5, and IL-17 A relative expression mRNA levels in 51 healthy human control and 51 UC tissue samples. As shown in Fig. 1, the relative mRNA expression level of ADAMTS-1 (p < 0.0001), ADAMTS-4 (p < 0.0001), ADAMTS-5 (p < 0.0001) and IL-17 A (p < 0.0001) were significantly higher in healthy human control tissue samples according to the UC tissue samples. Also, the relative mRNA expression levels of fold change were found at 10.9, 2.9, 19.3, and 9.3 for ADAMTS-1, -4, -5, and IL-17 A in the UC colon tissue samples according to the healthy human control tissue samples, respectively (Fig. 1).

**Fig. 2:**
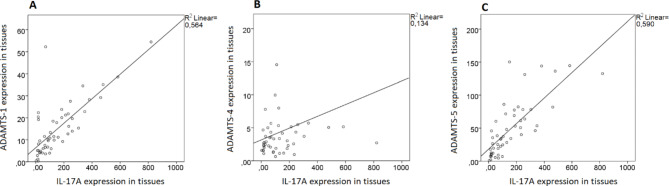
Correlation of ADAMTS-1, 4, 5, and IL-17 A expression in UC tissues. (**A**) ADAMTS-1 expression in tissues had positive correlation with tissues IL-17 A expression, *p* < 0.0001. (**B**) ADAMTS-4 expression in tissues had positive correlation with tissues IL-17 A expression, *p* = 0.008. (**C**) ADAMTS-5 expression in tissues had positive correlation with tissues IL-17 A expression, *p* < 0.0001

Also, to confirm whether the ADAMTS-1, -4, -5, and IL-17 A expression in UC tissues is consistent with each other in UC patients, correlation analysis was carried out for the expression of ADAMTS-1, -4, -5, and IL-17 A in matched tissues specimens. As a result, a strong positive correlation was found between transcript expressions of the ADAMTS-1 and IL-17 A (r^2^ = 0.564) (Fig. 2A). Also, the same manner was found between transcript expressions of the ADAMTS-5 and IL-17 A (r^2^ = 0.590) (Fig. 2C). On the other hand, a weak positive correlation was found between transcript expressions of the ADAMTS-4 and IL-17 A (r^2^ = 0.134) (Fig. 2B). The pearson correlation coefficients of ADAMTS-1, -4, and − 5 mRNA expression according to the IL-17 A mRNA expression were 0.751 (p < 0.0001), 0.366 (p = 0.008), and 0.768 (p < 0.0001), respectively.

**Immunohistochemical analysis of ADAMTS-1, ADAMTS-4, ADAMTS-5, and IL-17 A protein in the colon tissues of healthy human control and those with ulcerative colitis**.

**Fig. 3:**
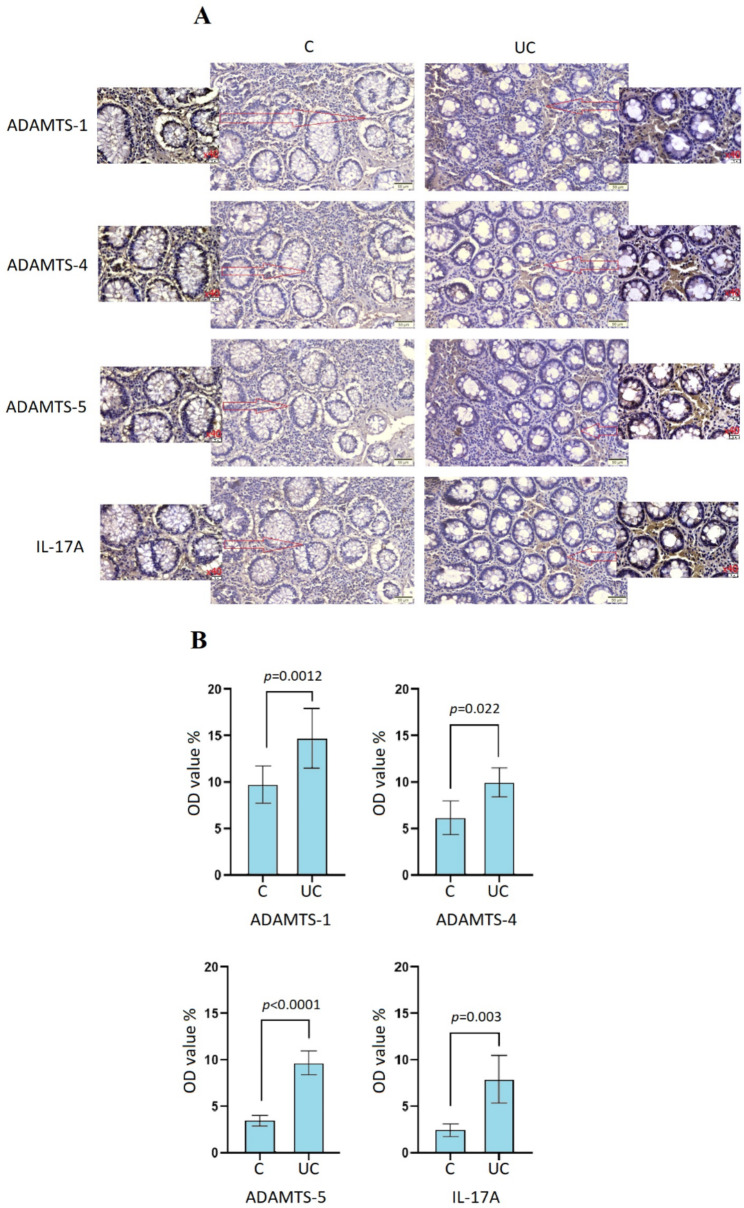
Immunohistochemical analysis of ADAMTS-1, -4, -5, and IL-17 A. (**A**) Immunohistochemical staining of ADAMTS-1, -4, -5, and IL-17 A in the healthy human control (C) and ulcerative colitis (UC) tissue sections (x20 magnification for main image and x40 magnification for outer image). (**B**) ADAMTS-1, -4, -5, and IL-17 A positive staining OD score of DAB-stained tissue sections in the healthy human control and ulcerative colitis tissue sections. The brownish staining shows the location of ADAMTS-1, -4, -5, and IL-17 A protein expression. The *t*-test represents a significant difference between healthy human control and UC tissue samples

Immunohistochemistry analysis was used to determine ADAMTS-1, -4, -5, and IL-17 A protein expression levels in healthy human control and UC tissue sections. The protein expression of ADAMTS-1, -4, -5, and IL-17 A was higher in UC tissue sections according to the healthy control sections (Fig. 3A). ADAMTS-1, -4, -5, and IL-17 A protein expressions in healthy control colon tissues appear weak staining in the gland boundary and lamina propria. However, ADAMTS-1, -4, -5, and IL-17 A protein expressions in certain regions of inflamed tissues of ulcerative colitis patients revealed to be significantly increased stained areas, especially in the lamina propria. The related areas of the tissue section was shown with different magnification in Fig. 3A [22, 23]. Also, the increased protein expression level (based on OD value of DAB stained areas) of ADAMTS-1 (*p* = 0.0012), ADAMTS-4 (*p* = 0.022), ADAMTS-5 (*p* < 0.0001), and IL-17 A (*p* = 0.003) were found significantly higher in the UC tissue sections according to the healthy control tissue sections (Fig. 3B).

### Serum level of the ADAMTS-1, ADAMTS-4, ADAMTS-5 and IL-17 A

**Fig. 4:**
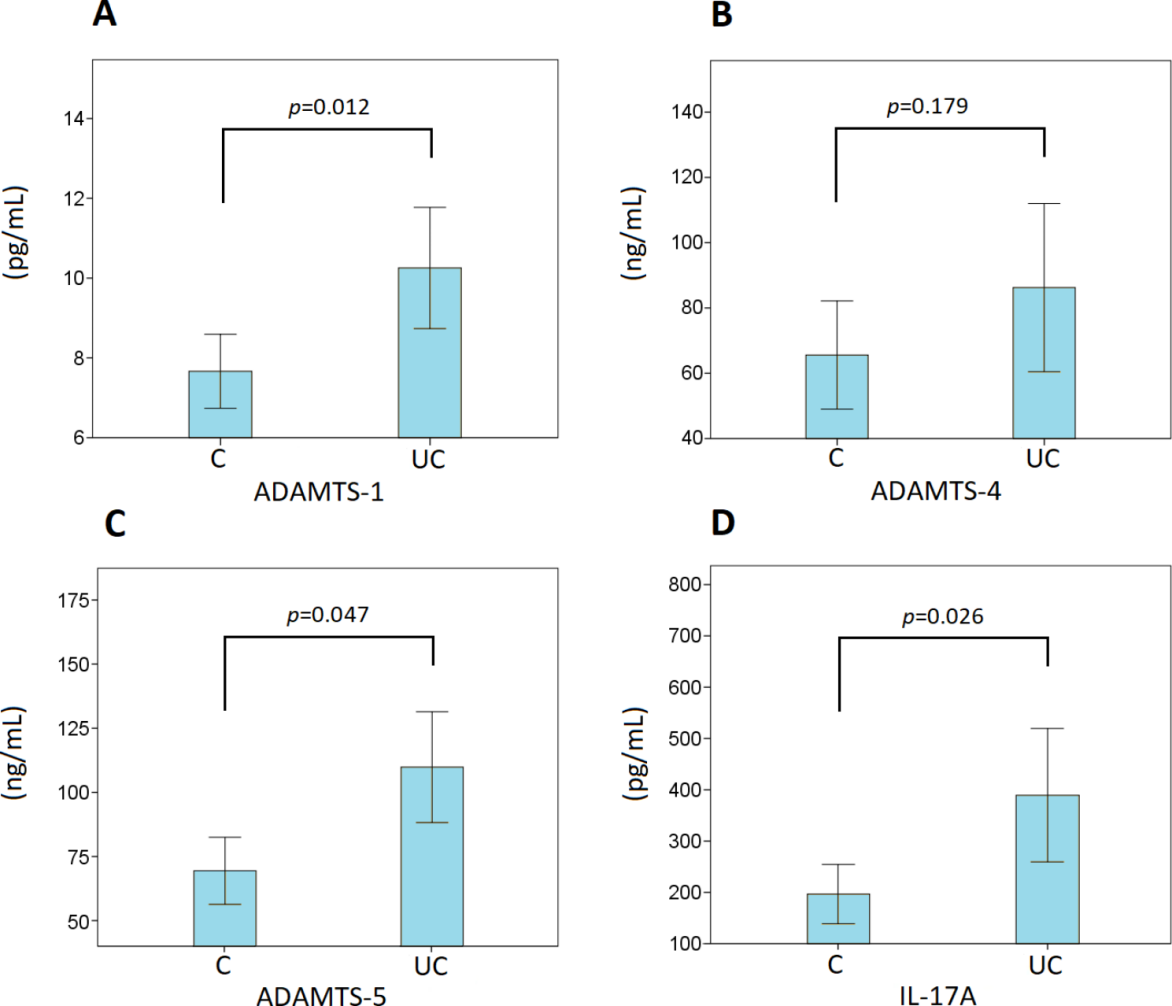
Serum levels of ADAMTS-1 (**A**), 4 (**B**), 5 (**C**), and IL-17 A (**D**) in the human healthy control (C) and ulcerative colitis (UC) groups

Also, serum ADAMTS-1, -4, -5, and IL-17 A levels were evaluated for the diagnostic potential in UC. The ELISA results reveal that ADAMTS-1, -4, -5, and IL-17 A serum levels in the UC group revealed significantly higher serum levels for ADAMTS-1, ADAMTS-5 and IL-17 A according to the control group (*p* = 0.012, *p* = 0.047 and *p* = 0.026, respectively) as shown in Fig. 3. However, no significant difference was found for the ADAMTS-4 serum level (*p* = 0.179) in the UC group according to the control group (Fig. 4).

**Fig. 5:**
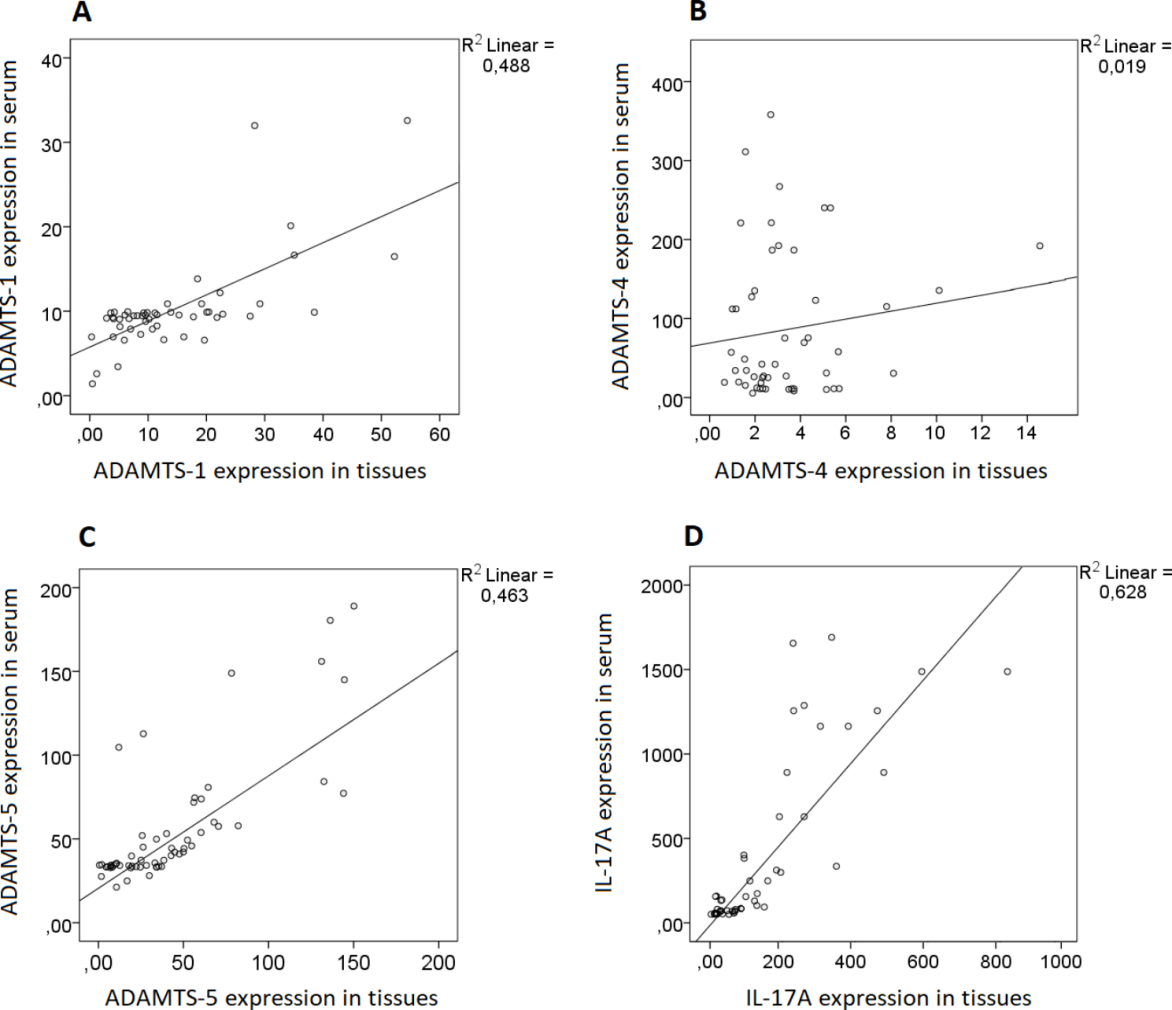
Correlation of ADAMTS-1, 4, 5, and IL-17 A expression in UC serum and UC tissues. (**A**) ADAMTS-1 expression in serum had positive correlation with tissues ADAMTS-1 expression, *p* < 0.0001. (**B**) ADAMTS-4 expression in serum had negative correlation with tissues ADAMTS-4 expression, *p* = 0.333. (**C**) ADAMTS-5 expression in serum had positive correlation with tissues ADAMTS-5 expression, *p* < 0.0001. (**D**) IL-17 A expression in serum had positive correlation with tissues IL-17 A expression, *p* < 0.0001

Also, to confirm whether the ADAMTS-1, -4, -5, and IL-17 A expression in UC tissues is consistent with the serum of UC patients, correlation analysis was carried out for the expression of ADAMTS-1, -4, -5, and IL-17 A in matched tissues and serum specimens. As a result, ADAMTS-1 (r^2^ = 0.488, *p* < 0.0001), ADAMTS-5 (r^2^ = 0.463, *p* < 0.0001) and IL-17 A (r^2^ = 0.628, *p* < 0.0001) expression values were positively correlated with tissue expression values (Fig. 5A, B and D). However, the ADAMTS-4 (r^2^ = 0.019, *p* = 0.333) expression value was not positively correlated with the tissue expression value (Fig. 5C).

**Fig. 6:**
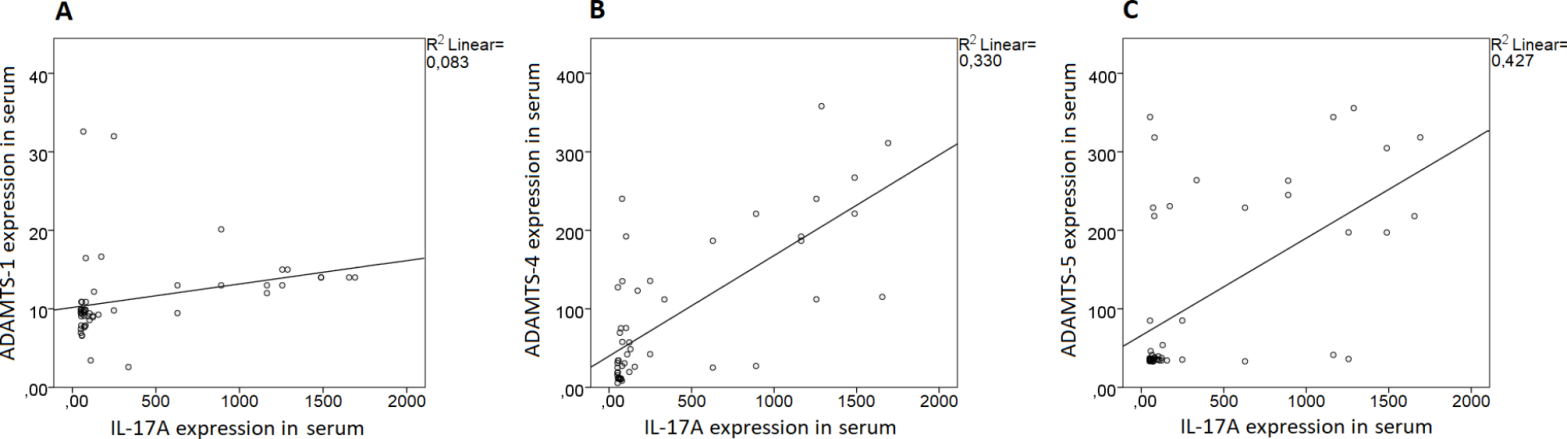
Correlation of ADAMTS-1, 4, 5, and IL-17 A expression in UC serum. (**A**) ADAMTS-1 expression in serum had positive correlation with serum IL-17 A expression, *p* = 0.040. (**B**) ADAMTS-4 expression in serum had positive correlation with serum IL-17 A expression, *p* < 0.0001. (**C**) ADAMTS-5 expression in serum had positive correlation with serum IL-17 A expression, *p* < 0.0001

Furthermore, the relationship of the ADAMTS-1, -4, -5, and IL-17 A expression in the serum was checked with correlation analysis in matched serum specimens of the UC patients. As a result, a weak positive correlation was found between expressions of the ADAMTS-1 and IL-17 A (r^2^ = 0.083) (Fig. 6A). Also, a moderate positive correlation was found between expressions of the ADAMTS-4 and IL-17 A (r^2^ = 0.330) (Fig. 6B). On the other hand, a strong positive correlation was found between expressions of the ADAMTS-5 and IL-17 A (r^2^ = 0.427, p < 0.0001) (Fig. 6C). The pearson correlation coefficients of ADAMTS-1, -4, and − 5 serum expression according to the IL-17 A serum expression were 0.289 (p = 0.040), 0.575 (p < 0.0001), and 0.726 (p < 0.0001), respectively.

## Discussion

Recent studies have shown evidence of IL-17 A cytokines and ADAMTS proteases’ relationship in the pathogenesis of many chronic inflammations. Unfortunately, the activity of IL-17 A cytokine involved in controlling inflammation during UC on ADAMTS members needs to be clearly understood. For this reason, the current first study aimed to improve understanding of how the expression of ADAMTS-1, -4, and − 5 are regulated by IL-17 A cytokine in UC tissues.

IL-17, also known as IL-17 A, is a key proinflammatory cytokine that links T-cell activation to neutrophil mobilization and activation. It is generally regarded as a bridge that connects innate and adaptive immunity and promotes inflammation and antimicrobial functions [24–26]. On the other hand, UC is caused by a complex interaction of environmental, genetic, and immunoregulatory factors [27, 28]. Although the effect of IL-17 in IBD is controversial, Ogawa et al. showed that IL-17 deficient or anti-IL-17 treated mice showed severe epithelial damage in the colon, indicating a protective function of IL-17 [29]. In another study, Sugihara et al. detected IL-17 mRNA expression in intestinal mucosa samples of patients with active UC and CD [30]. The data presented in this study demonstrated a statistically significant increase in ADAMTS-1, -4, -5, and IL-17 A mRNA expression levels in the UC colon tissue samples according to the control tissue samples (p < 0.0001, p < 0.0001, p < 0.0001, and p < 0.0001, respectively). While the highest mRNA expression level was found in ADAMTS-5 in the examined UC tissue samples, the increase in the mRNA expression level of ADAMTS-4 was lower than in other targeted genes in UC tissue samples according to the control tissue samples.

Although there is a positive correlation between the increase in the mRNA expression of the ADAMTS-1, -4, and − 5 genes and the increase in the IL-17 A mRNA expression level, this situation was found to be weaker for the ADAMTS-4. On the other hand, it is generally assumed that an increase in mRNA expression leads to an increase in protein levels, but it is not always the case. Several factors can influence the correlation between mRNA expression and protein levels, such as post-transcriptional regulation, translation efficiency, protein degradation, and regulatory mechanisms [31, 32]. For this reason, the expression of genes at the mRNA level not result in a proportional increase in protein levels of this study. In immunohistochemistry evaluations on tissues, it was observed that there was a significant increase in ADAMTS-1 (p = 0.0012), -4 (p = 0.0022), -5 (p < 0.0001), and IL-17 A (p = 0.003) protein expression levels in UC tissues compared to control tissues, as well as mRNA expression levels. Although the literature on the expression of ADAMTS-1, -4, and − 5 and IL-17 A proteins in tissue is limited, it has been reported that ADAMTS-1, -4, and − 5 proteins are expressed in the mucosal layer and also in intestinal cells between glands [33, 34]. In our study, low levels of staining for ADAMTS-1, -4, -5, and IL-17 A proteins were observed in healthy colon, mainly in the glandular cells and in intestinal cells between glands. On the other hand, strong levels of staining for ADAMTS-1, -4, -5, and IL-17 A proteins were observed in the UC colon, mainly in the intestinal cells between glands. Also, a decrease was found in glandular lumen diameters, and an increase in their number was observed in UC tissue sections compared to control sections. Similar to the mRNA expression levels, the highest protein expression fold change level was found in ADAMTS-5 in the UC tissue samples examined. In contrast, the increases in the protein expression fold change level of ADAMTS-4 were more moderate than other protein levels.

A significant increase was determined in serum expression levels of ADAMTS-1, -5, and IL-17 A in serum samples from UC patients compared to control serum samples. On the other hand, there was no significant increase in ADAMTS-4 expression levels in UC serum samples compared to control serum samples and there was no significant correlation between matched UC tissues and serum specimens for ADAMTS-4 expression. However, a moderate correlation was found between the ADAMTS-4 and IL-17 A expression in UC serum specimens. In the correlation analysis, a positive correlation was found between serum and tissue expression of ADAMTS-1, -5, and IL-17 A. Also, a positive correlation was found for the ADAMTS-1, -4, and − 5 serum expression according to the IL-17 A serum expression. However, a strong correlation was only found between the ADAMTS-5 and IL-17 A serum expression. As a reference for the IL-17 serum expression in UC patients, a study on the Chinese population reported significantly higher expression levels of IL-17 in serum and IL-17R levels in the mucosa in UC patients [34].

## Conclusion

This study obtained a higher correlation level between ADAMTS-5 and IL-17 A according to ADAMTS-1 and − 4. In addition, increasing ADAMTS-5 mRNA and protein expression was more significantly associated with increasing Il-17 mRNA and protein expression in tissues and serum. In addition, the expression rate of ADAMTS-5 in tissues and serum was higher according to ADAMTS-1 and − 4. Taken together, these findings suggest that IL-17 A may promote the breakdown of the intestinal wall in ulcerative colitis by stimulating the high expression of ADAMTS-5. However, more research is needed to fully understand the relationship between these molecules and their roles in the development of the UC.

## Data Availability

The datasets used and analyzed during the current study are available from the corresponding author on reasonable request.

## Abbreviations

ADAMTS: A disintegrin and metalloproteinase with thrombospondin motifs
DAB: Diaminobenzidine
ELISA: Enzyme-linked immuno sorbent assay
IHC: Immunohistochemistry
IL-17 A: Interleukin 17 A
OD: Optical density
RT-qPCR: Reverse transcription quantitative real-time polymerase chain reaction
UC: Ulcerative colitis
